# iRGvalid: A Robust *in silico* Method for Optimal Reference Gene Validation

**DOI:** 10.3389/fgene.2021.716653

**Published:** 2021-08-04

**Authors:** Zhongxu Zhu, Keqin Gregg, Wenli Zhou

**Affiliations:** XYZ Laboratory, Austin, TX, United States

**Keywords:** reference gene, gene expression, cancer, reference gene selection, reference gene validation, *in silico* reference gene selection, *in silico* reference gene validation

## Abstract

**Background:**

Appropriate reference genes are critical to accurately quantifying relative gene expression in research and clinical applications. Numerous efforts have been made to select the most stable reference gene(s), but a consensus has yet to be achieved. In this report, we propose an *in silico* reference gene validation method, iRGvalid, that can be used as a universal tool to validate the reference genes recommended from different resources so as to identify the best ones without a need for any wet lab validation tests.

**Methods:**

iRGvalid takes advantage of high throughput gene expression data and is built on a double-normalization strategy. First, the expression level of each individual gene is normalized against the total gene expression level of each sample, followed by a target gene normalization to the candidate reference gene(s). Linear regression analysis is then performed between the pre- and post- normalized target gene across the whole sample set to evaluate the stability of the reference gene(s), which is positively associated with the *Pearson* correlation coefficient, Rt. The higher the Rt value, the more stable the reference gene. We applied iRGvalid to 14 candidate reference genes to validate and identify the most stable reference genes in four cancer types: lung adenocarcinoma, breast cancer, colon adenocarcinoma, and nasopharyngeal cancer. The stability of the reference gene is evaluated both individually and in groups of all possible combinations.

**Results:**

Highly stable reference genes resulted in high Rt values regardless of the target gene used. The highest stability was achieved with a specific combination of 3 to 6 reference genes. A few genes were among the best reference genes across the cancer types studied here.

**Conclusion:**

iRGvalid provides an easy and robust method to validate and identify the most stable reference gene or genes from a pool of candidate reference genes. The inclusivity of large expression data sets as well as the direct comparison of candidate reference genes makes it possible to identify reference genes with universal quality. This method can be used in any other gene expression studies when large cohorts of expression data are available.

## Introduction

As an important biomarker source, gene expression has been one of the major focuses of cancer genome studies. Appropriate reference genes are critical to accurately quantifying relative expression levels. Numerous studies have been performed to identify the most stable reference genes in different tissues or cells (Vandesompele et al., 2002; Andersen et al., 2004; Pfaffl et al., 2004), but a consensus has yet to be achieved (Wang et al., 2012; Jacob et al., 2013). The problem becomes even more prominent in cancer studies due to intrinsically unstable gene expression and the heterogeneity of cancer tissues.

Aggregated and publicly available data generated from whole genome expression studies have been used as a resource to search for more widely and stably expressed reference genes. However, there is still no consensus among researchers. Popovici et al. (2009) used microarray data from 10 cohorts of breast cancer studies and identified the 50 most stably expressed genes. Tilli et al. (2016) later obtained 10 novel reference genes from 6 breast cancer cell lines using both transcriptome and microarray data from several databases. The two sets of reference genes were not agreeable. In 2019, two groups independently published their work on the selection of pan-cancer reference genes using RNA-Seq data from hundreds of cancerous and matched normal tissue samples across all cancer types, primarily in the Cancer Genome Atlas (TCGA) database (Jo et al., 2019; Krasnov et al., 2019). One group (Jo et al., 2019) found 32 novel genes that had the best stabilities, among which *HNRNPL, PCBP1*, and *RER1* were claimed as the most suitable reference genes for all cancer types, whereas another group (Krasnov et al., 2019) analyzed 12 cancer types, ranked the most stably expressed genes, and recommended *SF3A1, CIAO1*, and *SFRS4* as the best reference genes for cancer studies.

The lack of consistency in reference gene recommendations puts the burden on researchers to pick the right reference gene(s) for their studies and makes it difficult to compare the results of different studies. Thus, researchers often have to perform wet lab validation tests to identify the best reference genes among recommended ones, which is time-consuming and labor-intensive. Moreover, the limited number of samples and lack of absolute quantification can cause some biases. By taking advantage of RNA-Seq data from the TCGA database, we developed an easy and robust *in silico* reference gene validation method, iRGvalid, and used this method to validate the reference genes recommended by two studies mentioned above (Jo et al., 2019; Krasnov et al., 2019) as well as those selected in-house from the TCGA database. The results presented here demonstrate that this method could be a useful tool for researchers to evaluate candidate reference genes and identify the most suitable ones for their studies without a wet lab validation.

## Materials and Equipment

### TCGA Data Collection

Gene expression datasets of 33 TCGA projects were downloaded using the TCGABiolinks (2.14.1) package (Colaprico et al., 2016). The workflow type parameter was set to “HTSeq – FPKM” in “TCGABiolinks” “GDCquery” function. For the sample selection, the TCGA barcodes were parsed. The tumor samples without matched normal samples were filtered out, and the paired normal and tumor tissue samples were selected. Additionally, 110 nasopharyngeal carcinoma (NPC) samples with raw RNA-Seq data (NCBI SRA study accession number, SRP115011) were used as an independent validation cohort (Leinonen et al., 2011; Zhang et al., 2017).

### Nasopharyngeal Carcinoma (NPC) Data Collection

A cohort of NPC RNA-Seq data was obtained from Zhang et al. (2017) (SRP11501). The sequencing adapters and low-quality reads were first filtered out from raw sequencing reads using FastP software (Chen et al., 2018). Clean reads were aligned to the reference genome (hg38, Ensembl gene annotation, Version 99) with STAR aligner (Dobin et al., 2013). Only unique reads were kept. Gene expression levels were quantified with SALMON software (Patro et al., 2017) based on alignment results.

### Methods

**FIGURE 1:**
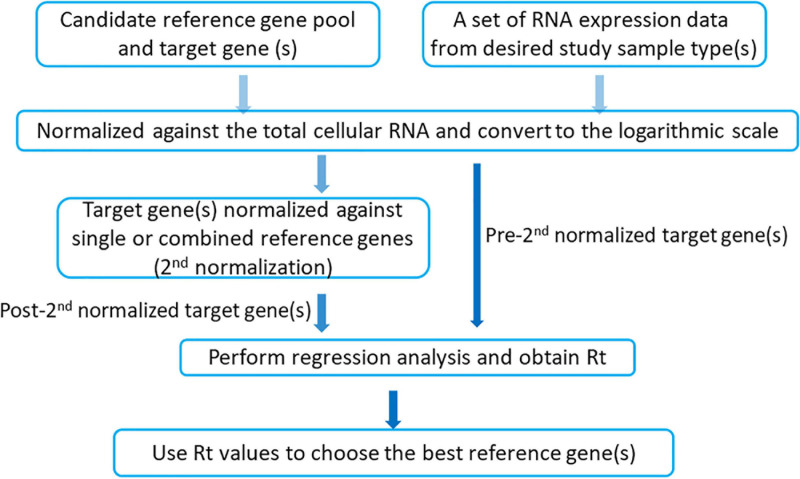
iRGvalid workflow.

The iRGvalid workflow is shown in Figure 1. A candidate reference gene pool selected from literatures and in-house studies is established first. A set of expression data that represents a study population is then chosen from a database. The measurement of expression level is converted from FPKM (Fragments Per Kilobase of transcript per Million) to TPM (Transcripts Per Million), followed by log2 transformation. Next, the target gene is normalized against a single or various combinations of candidate reference genes using the formula Log_2_(TPM + 1)_target_-Log_2_(TPM + 1)_ref_ for a single reference gene and the arithmetic mean of Log_2_(TPM + 1)_ref_ for a combinations of reference genes. Finally, the *Pearson* correlation coefficient Rt is calculated via regression analysis of the pre- and post- normalized target gene by R package “stats,” where “t” stands for target gene. The higher the Rt, the more stable and better the reference gene or combined reference genes in regards to the target gene. For a perfect reference gene(s), Rt should be close to 1 and target insensitive. In the following section, we provide examples that apply iRGvalid to validate candidate reference genes for lung adenocarcinoma (LUAD), breast invasive carcinoma (BRCA), colon adenocarcinoma (COAD), and nasopharyngeal carcinoma (NPC).

### Interactive Online Application

An online application was created using the Rstudio’s Shiny framework. It can be found at https://wlake.shinyapps.io/iRGvalid/ where users can retrieve the analysis results reported in this manuscript, and perform iRGvalid analysis by providing a specific target gene and reference gene(s). Further instruction is provided in the Supplementary Document.

## Results

### Select Candidate Reference Genes

The candidate reference genes used in this study include genes recommended by two published studies (Jo et al., 2019; Krasnov et al., 2019; Table 1), and ones selected in-house. The in-house reference gene selection was based on a gene expression variation analysis of 22 cancer types with 679 paired cancerous and normal sample sets from 33 TCGA projects. The final ranking of the 10 best candidate reference genes (Table 1, right column) were selected based on the following criteria: (1) The coefficient variance (CV) was less than 8% in both normal and cancerous tissues of all cancer types; (2) The expression level was high, i.e., Log_2_(TPM) > 7; (3) The difference in expression levels between normal and cancerous tissues was minimal, i.e., |Log2(TPM_cancer_)-Log2(TPM_normal_)| < 0.1; and (4) The correlation coefficient between any pair of candidate reference genes was less than 0.5. As shown in Table 1, there was no consensus among the reference genes identified in any of the studies, even though they were all obtained primarily using the TCGA databases. To perform the reference gene validation with iRGvalid, we selected a candidate reference gene pool consisting of six highly recommended reference genes from the aforementioned studies: *HNRNPL*, *PCBP1*, *RER1*, *SF3A1*, *CIAO1*, and *SFRS4*, and six genes from this study: *CNBP*, *MYL12B*, *UBC*, *TMBIMG*, *RPS27*, and *EIF1* (Table 1 in bold). We also added a commonly used reference gene in cancer studies, *GAPDH*, and a randomly selected non-reference gene, *HEY1*, for a total of 14 pan-cancer candidate reference genes.

**TABLE 1:** Candidate reference genes from different studies.

**Rank**	**Krasnov et al.**	**Jo et al.**	**This study**
1	*MBTPS1*	***HNRNPL***	***CNBP***
2	*HNRNPA0*	***PCBP1***	*RPL36AL*
3	***SF3A1***	*PFDN1*	***EIF1***
4	*SF3B2*	***RER1***	***MYL12B***
5	*GGNBP2*	*RNF10*	***UBC***
6	*HNRNPUL2*	*SNX17*	*RPS12*
7	*SFRS3*	*EMC4*	***TMBIM6***
8	*RTF1*	*FAM32A*	***RPS27***
9	***CIAO1***	*HNRNPC*	*RPL11*
10	*TM9SF3*	*IST1*	*RNF167*
11	*PRPF8*	*MRPL43*	
12	*GTF2F1*		
13	***SFRS4***		
14	*SARS*		
15	*ARIH1*		
16	*TEX261*		
17	*VCP*		
18	*XRCC5*		
19	*VPS4A*		
20	*KPNA6*		

*iRGvalid were performed for the bolded ones.*

### Validate Single Candidate Reference Genes

**FIGURE 2:**
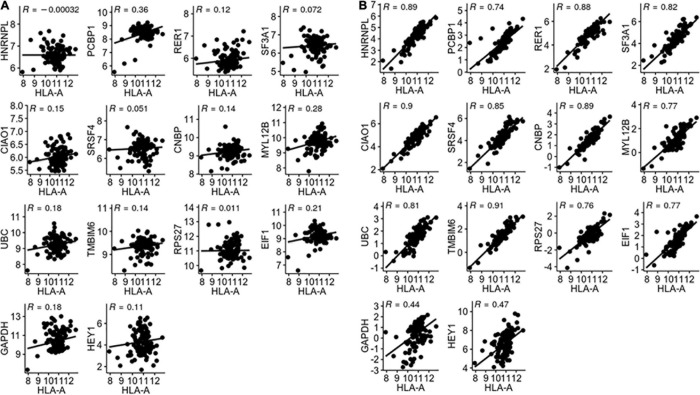
**(A)** Correlation between HLA-A and reference gene expression. **(B)** Correlation between pre- and post-normalized HLA-A gene expression.

We first validated the candidate reference genes individually in LUAD using *HLA-A* as a target gene. *HLA-A* is known to play an important role in cancer development and immune response (Garrido, 2019). A total of 57 pairs of LUAD and corresponding normal tissue RNA-Seq data were obtained from TCGA. The TPM of the *HLA-A* gene and 14 reference genes were calculated for each of the 57 pairs of samples. By definition, the reference gene cannot have any correlation with the target gene. As shown in Figure 2A, none of the candidate reference genes had any significant correlation with *HLA-A*. *HLA-A* was normalized for each of the candidate reference genes, and Rt between the pre- and post- normalization Log (TPM_HLA–A_ + 1) values was obtained for each candidate reference gene (Figure 2B). *TMBIM6* and *CIAO1* had the highest Rt values (0.911 and 0.903, respectively). We then repeated the validation analysis with other two target genes, *HIF1A* and *ERBB3*, both of which have expressions associated with lung cancer progress and prognosis (Chen et al., 2007; Yang et al., 2016). The Rt of the three target genes normalized with each of the candidate reference genes is given in Table 2. *TMBIM6*, *CIAO1*, and *CNBP* had the top 3 Rt values for all three target genes. We also averaged the Rt values of the three target genes for each individual candidate reference gene. *TMBIM6*, *CIAO1*, and *CNBP* had the highest Rt values and thus can be considered as the three best single reference genes in LUAD.

**TABLE 2:** Target gene Rt and average Rt of each single reference gene in four types of cancers.

**Cancer types**		**Candidate Reference Gene**													
		***HNRNPL***	***PCBP1***	***RER1***	***SF3A1***	***CIAO1***	***SRSF4***	***CNBP***	***MYL12B***	***UBC***	***TMBIM6***	***RPS27***	***EIF1***	***GAPDH***	***HEY1***
LUAD	*HLA-A*	0.886	0.738	0.879	0.822	0.903	0.846	0.889	0.775	0.809	0.911	0.757	0.767	0.436	0.473
	*ERBB3*	0.894	0.828	0.906	0.833	0.924	0.87	0.929	0.879	0.86	0.931	0.815	0.859	0.557	0.650
	*HIF1A*	0.904	0.849	0.911	0.876	0.931	0.898	0.928	0.912	0.868	0.955	0.84	0.885	0.19	0.798
	Average	0.895	0.805	0.899	0.844	0.919	0.871	0.915	0.855	0.846	0.932	0.804	0.837	0.394	0.640
BRCA	*HER2*	0.887	0.800	0.895	0.820	0.920	0.864	0.937	0.871	0.841	0.930	0.806	0.842	0.473	0.654
	*MYBL2*	0.991	0.974	0.989	0.979	0.992	0.982	0.987	0.983	0.979	0.992	0.964	0.979	0.908	0.940
	*MMP11*	0.992	0.983	0.993	0.986	0.994	0.988	0.992	0.987	0.986	0.995	0.976	0.986	0.935	0.947
	Average	0.957	0.919	0.959	0.928	0.969	0.945	0.972	0.947	0.935	0.972	0.915	0.936	0.772	0.847
COAD	*NOTCH2*	0.850	0.794	0.886	0.752	0.885	0.783	0.904	0.892	0.774	0.913	0.868	0.840	0.465	0.586
	*BRCA1*	0.925	0.860	0.916	0.865	0.942	0.884	0.934	0.931	0.877	0.954	0.858	0.900	0.276	0.768
	*PDC*	0.811	0.896	0.785	0.795	0.834	0.705	0.860	0.810	0.803	0.882	0.770	0.816	0.520	0.481
	Average	0.862	0.850	0.862	0.804	0.887	0.791	0.899	0.878	0.818	0.916	0.832	0.852	0.420	0.612
NPC	*HLA-A*	0.881	0.789	0.845	0.688	0.903	0.871	0.893	0.852	0.83	0.814	0.870	0.887	0.780	0.604
	*ANXA1*	0.968	0.945	0.963	0.910	0.972	0.964	0.966	0.964	0.958	0.943	0.960	0.969	0.888	0.836
	*FNDC3B*	0.869	0.800	0.869	0.585	0.897	0.878	0.877	0.878	0.849	0.816	0.887	0.889	0.575	0.515
	Average	0.906	0.845	0.892	0.728	0.924	0.904	0.912	0.898	0.879	0.858	0.906	0.915	0.748	0.652

We next validated the 14 candidate reference genes in 121 pairs of BRCA samples and 41 pairs of COAD samples, respectively. Three clinically significant and highly studied genes were selected as target genes for each cancer type: *HERE2*, *MYBL2*, and *MMP11* for BRCA (Paik et al., 2004) and *NOTCH2*, *BRCA1*, and *PDC* for COAD (Abdul Aziz et al., 2016). None of the target genes had significant correlations with the candidate reference genes (data not shown). As shown in Table 2, all three target genes in breast cancer had a high correlation between pre- and post-normalization, indicating that all of the reference genes examined here had good stability across breast cancerous tissues and their paired normal tissues. The top average Rt values for the three target genes in both BRCA and COAD are also *TMBIM6*, *CIAO1*, and *CNBP* (Table 2).

To illustrate the robustness of the iRGvalid method, we further tested the candidate reference genes on an independent cohort of NPC RNA-Seq data. This set of data was not part of the TCGA data used to obtain the candidate reference genes. We again selected three target genes that play important roles in NPC progress and prognosis: *ANXA1*, *FNDC3B*, and *HLA-A*. The best single reference genes for the NPC set were *CIAO1, EIF1*, and *CNBP*.

*GADPH* and *HEY1* had the worst Rt values in all target genes and cancer types examined.

### Validate the Combined Reference Genes

**FIGURE 3:**
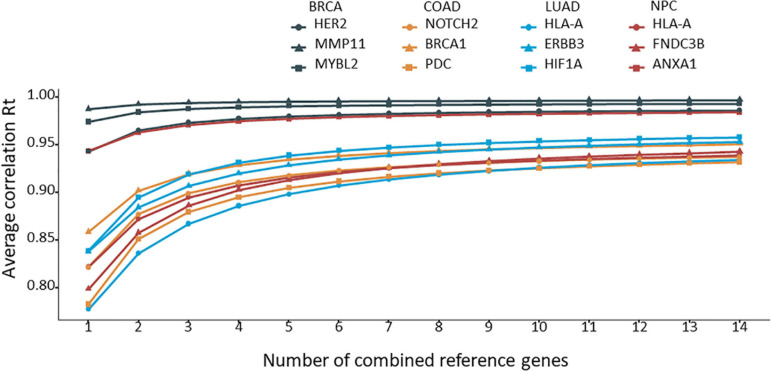
The average correlation coefficient (Rt) for each number of combined reference genes. The target gene was normalized to the corresponding number of combined reference genes, and the average Rt was calculated and plotted.

**FIGURE 4:**
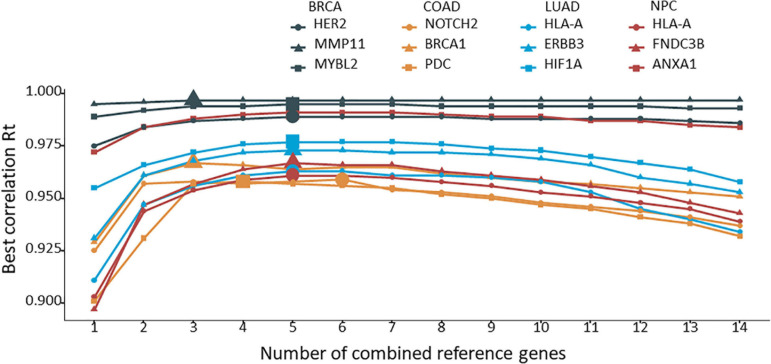
The best correlation coefficient (Rt) for each number of combined reference genes. The target gene was normalized to the corresponding number of combined reference genes, and the best Rt was identified from the various combinations and plotted.

The 12 target genes in their corresponding cancer types described above were normalized with all possible combinations of 2 to 14 reference genes. Rt was calculated for each normalization and ranked for each target gene. The average Rt value of each number of combined reference genes was calculated and plotted for each target gene. As shown in Figure 3, by increasing the number of reference genes, the average Rt became higher, indicating an improved overall reference gene stability. However, average Rt could undermine some highly stable reference gene combinations. To test this idea, the best Rt for each number of combined reference genes was evaluated. The results clearly demonstrated that more was not necessarily better (Figure 4). The best Rt was obtained with 3 to 6 combined reference genes. Once the Rt reached its peak, adding more reference genes made the combined reference genes less stable. This was true for all four cancer types examined.

### The Best Combination of Reference Genes

The best combined reference genes for each target gene in each cancer type are listed in Table 3. In the BRCA and NPC data sets, all three target genes had the same best reference gene combination: *CIAO1*, *CNBP*, *HNRNPL*, *RER1*, and *SRSF4* in BRCA, and *EIF1*, *MYL12B*, *RER1*, *RPS27*, and *SRSF4* in NPC. In LUAD, two target genes had the same set of best reference gene combination: *CIAO1*, *CNBP*, *RER1*, *SRSF4*, and *TMBIM6*. More variations were seen in COAD. Three reference genes, *RER1*, *RPS27*, and *SRSF4*, were present in almost all of the best combinations.

**TABLE 3:** Rt of the best combined reference genes for each target gene in each cancer type.

**Cancer Type**	**Target Gene**	**Rt**	**Number of combined reference genes**	**Panel**
BRCA	*HER2*	0.989	5	*CIAO1, CNBP, HNRNPL, RER1, SRSF4*
	*MMP11*	0.997	3 to 5	*CIAO1, CNBP, HNRNPL, RER1, SRSF4*
	*MYBL2*	0.995	5	*CIAO1, CNBP, HNRNPL, RER1, SRSF4*
COAD	*BRCA1*	0.967	3	*CIAO1, HNRNPL, RER1*
	*PDC*	0.958	4	*PCBP1, RER1, RPS27, UBC*
	*NOTCH2*	0.959	6	*CIAO1, CNBP, HNRNPL, MYL12B, RER1, UBC*
LUAD	*ERBB3*	0.973	5	*CIAO1, CNBP, RER1, SRSF4, TMBIM6*
	*HIF1A*	0.977	5	*MYL12B, RER1, RPS27, SRSF4, TMBIM6*
	*HLA-A*	0.963	5	*CIAO1, CNBP, RER1, SRSF4, TMBIM6*
NPC	*ANXA1*	0.991	5	*EIF1, MYL12B, RER1, RPS27, SRSF4*
	*FNDC3B*	0.967	5	*EIF1, MYL12B, RER1, RPS27, SRSF4*
	*HLA-A*	0.961	5	*EIF1, MYL12B, RER1, RPS27, SRSF4*

While Table 3 lists one best combination of reference genes for each target gene, some target genes had more than one set of best combined reference genes. Notably, *MMP11* had a total of 56 best sets of reference genes with 3 to 5 genes in each (data not shown). Interestingly, the best set of reference genes for each target gene was not necessarily a combination of the top single reference genes.

## Discussion

In this study, we presented an *in silico* method, iRGvalid, that takes advantage of massive RNA-Seq data to validate candidate reference genes for their stability so as to identify the best ones without needing a wet lab validation. A reference gene is solely used for normalization in gene expression studies (Bustin et al., 2009), and the normalization should not change the relative relations of a gene among all of the individual samples if the reference gene is good. The iRGvalid method is built upon this fundamental principle. The method is straightforward and intuitive. The *Pearson* correlation coefficient between the pre- and post- normalized target gene is used to measure the stability of a reference gene or a combination of reference genes. In this report, we only selected three target genes in each of the cancer types to validate the candidate reference genes. However, the number of target genes is not limited in the iRGvalid validation procedure. A stable reference gene generates a high correlation coefficient and should be target insensitive. As far as we are aware, this is the first *in silico* reference gene validation method that involves the active normalization of target gene(s) in evaluating the stability of reference gene(s). We tested the TCGA RNA-Seq data here, but the method can be applied to any other formats of expression data sets.

The validation results of the single versus group reference genes demonstrated that an appropriately selected combination of reference genes works better than a single reference gene. The best combination can be achieved with 3–6 genes and need not be unique for a particular target gene. We expect that the best combination of reference genes may not be unique for a particular study either. Nevertheless, an ideal set of reference genes should be stable across all samples in a cohort regardless of the target gene selected. Our data demonstrated this principle well in BRCA, LUAD, and NPC, but not so much in COAD. One explanation could be that COAD had the smallest sample size in this study: 82 compared to 111, 114, and 224 for NPC, LUAD, and BRCA, respectively. The larger the sample size, the truer the *Pearson* correlation coefficient, and the better the chance of finding a true set of stable reference genes. The analysis can be performed again when more COAD data becomes available. It is also likely that the candidate reference gene pool used in this study was not optimal for COAD.

Of the 14 candidate reference genes used in this study, 12 were selected from pan-cancer data sets and claimed to be the best reference genes across all types of cancers in each specific study. However, our validation using iRGvalid showed that the best universal reference genes are *RER1*, *RPS27*, and *SRSF4*. Each of these three genes came from a different candidate reference gene source, demonstrating the importance and necessity of uniting the candidate reference gene pools and validating them under the same condition, i.e., using iRGvalid. The current claim of pan-cancer reference genes may be preemptive, though. While more data will better validate the stability of the reference genes, it is also entirely likely that the best reference genes are cancer-type specific due to different cancer etiologies across organs and tissues.

The *HEY1* gene encodes a helix-loop-helix transcription factor. It interacts with other transcription factors and regulates a variety of cellular activities (Weber et al., 2014). Deregulation and aberrant expression of *HEY1* have been documented in diverse malignancies, including colorectal cancer. It by no means should be a reference gene. In this study, it performed poorly when judged by the Rt values of target genes. Unsurprisingly, *GAPDH* is also not a good reference gene. Although it has been one of the most frequently used reference genes, *GAPDH* has shown to be less stably expressed than many other commonly used reference genes in cancers (Jacob et al., 2013; Jo et al., 2019; Krasnov et al., 2019). The presence of *GAPDH* pseudogenes could potentially complicate any quantifications (Sun et al., 2012).

iRGvalid allows one to systematically evaluate and select the best reference genes when there are large cohorts of expression data. We have provided several examples of using the method. By no means was our intention to choose reference genes for any particular cancer studied in this report. Instead, we have illustrated an effective method for doing so. iRGvalid can also help to check whether the reference gene(s) used are stable against newly identified, differentially expressed genes. The effectiveness of the method depends on the quality of candidate reference genes and the validation sample size. It is less effective when the validation sample size is small, which is a major limitation of the method. With the increasing size of the RNA-Seq database, the stable and optimal number of reference genes can be more reliably determined in any type of cancer or tissue using iRGvalid.

## Data Availability Statement

The datasets analyzed during the current study are available in the TCGA repository (https://cancergenome.nih.gov/) and the results are available in the online application (https://wlake.shinyapps.io/iRGvalid/).

## Author Contributions

WZ contributed to conception of the study. WZ, ZZ, and KG contributed to the design of the study. ZZ performed all the analysis and built the online application. WZ wrote the first draft of the manuscript. All authors contributed to manuscript revision, read, and approved the submitted version.

## Conflict of Interest

WZ and KG were employed by XYZ Laboratory. ZZ declares that the research was conducted in the absence of any commercial or financial relationships that could be construed as a potential conflict of interest.

## Publisher’s Note

All claims expressed in this article are solely those of the authors and do not necessarily represent those of their affiliated organizations, or those of the publisher, the editors and the reviewers. Any product that may be evaluated in this article, or claim that may be made by its manufacturer, is not guaranteed or endorsed by the publisher.

## Abbreviations

BRCA: breast invasive carcinoma
COAD: colon adenocarcinoma
iRGselect: *in Silico* reference gene validation and selection
LUAD: lung adenocarcinoma
NPC: nasopharyngeal carcinoma
TCGA: the Cancer Genome Atlas
TPM: Transcripts Per Million.
