# Supplementary material for: iRGvalid: A Robust in silico Method for Optimal Reference Gene Validation
Source: Front Genet. 2021 Aug 4;12:716653. doi: 10.3389/fgene.2021.716653 (PMC8372526; doi:10.3389/fgene.2021.716653)

Introduction to the online application

An interactive online application has been created with a parameter panel on the left and a results panel on the right. Currently, it has two major functions. First, it provides all of the data and results mentioned in this manuscript. Users can specify a dataset (BRCA, COAD, LUAD, or NPC) and choose a range of Rt values on the left panel. The relevant results will be displayed on the right panel in real-time (an example is illustrated in Figure S1A). Secondly, it provides a service for users to perform iRGvalid analysis. Users can specify a target gene and reference gene(s), as well as select one or more TCGA datasets. The expression of the target gene will be normalized against all combinations of the candidate gene(s), and the Rt value will be displayed (as shown in Figure S1B). All combinations of the candidate gene(s) will also be analyzed iteratively. The best combination of reference genes with the highest Rt value will be shown at the top of the results analysis. Furthermore, all of the data, including the expression levels of reference genes and the expression levels pre- and post-normalization, will be also shown in the web application.

Figure S1

A

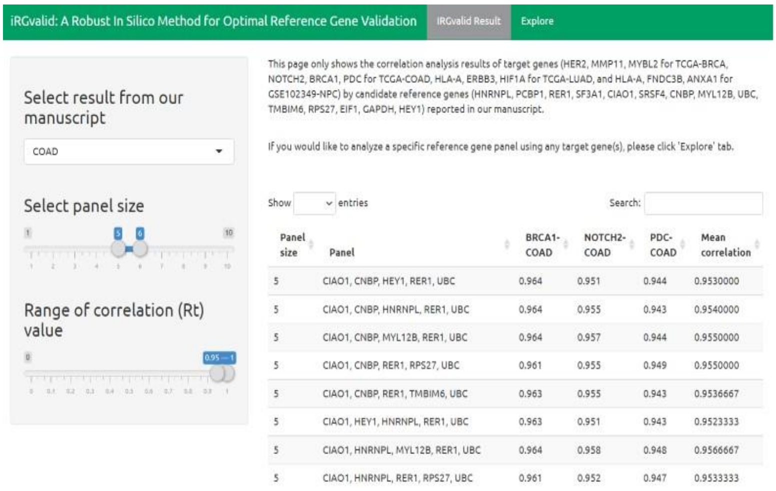

B

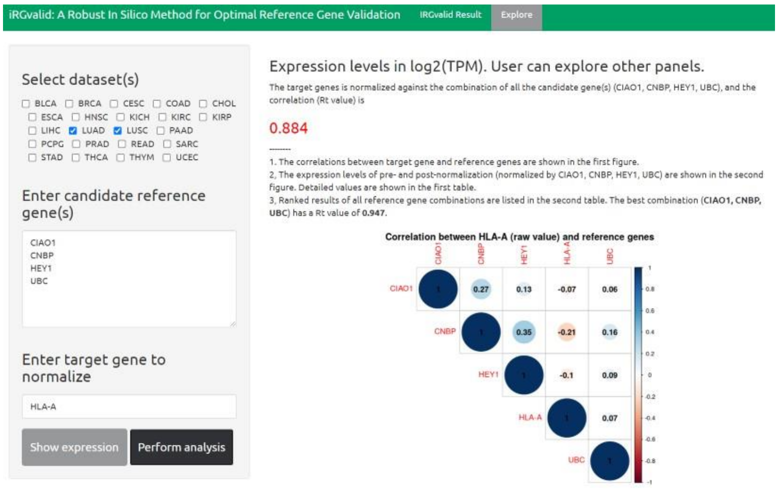

Supplement: Supplementary file 1 [file Presentation_1.pdf]
